# Clinical and Prognostic Significance of Preoperative Plasma Fibrinogen Levels in Patients with Operable Breast Cancer

**DOI:** 10.1371/journal.pone.0146233

**Published:** 2016-01-22

**Authors:** Yu Mei, Song Zhao, Xiaofei Lu, Haixia Liu, Xiangyi Li, Rong Ma

**Affiliations:** 1 Department of Breast Surgery, Qilu Hospital of Shandong University, Jinan, Shandong, PR China; 2 Department of Breast Surgery, Jinan Maternity and Child Care Hospital, Jinan, Shandong, PR China; 3 Department of General Surgery, Jinan Central Hospital Affiliated to Shandong University, Jinan, Shandong, PR China; 4 Department of Pathology, Jinan Maternity and Child Care Hospital, Jinan, Shandong, PR China; 5 School of Public Health, Shandong University, Jinan, Shandong, PR China; University of North Carolina School of Medicine, UNITED STATES

## Abstract

**Purpose:**

Elevated plasma fibrinogen levels are associated with tumor progression and poor outcomes in different cancer patients. The objective of this study was to investigate the clinical and prognostic value of preoperative plasma fibrinogen levels in patients with operable breast cancer.

**Methods:**

Two hundred and twenty-three patients diagnosed with breast cancer were retrospectively evaluated in this study. Plasma fibrinogen levels were examined before treatment and analyzed along with patient clinicopathological parameters, disease-free survival (DFS) and overall survival(OS). Both univariate and multivariate analyses were performed to identify the clinicopathological parameters associated with DFS and OS.

**Results:**

Elevated preoperative plasma fibrinogen levels were directly associated with age of diagnose (≤47 vs. >47, *p*<0.001), menopause (yes vs. no, p<0.001), tumor size (T1&T2 vs.T3&T4, *p* = 0.033), tumor stage (Ⅰvs.Ⅱvs.Ⅲ, *p* = 0.034) and lymph node involvement (N = 0 vs. 1≤N≤3 vs. N≥4, *p*<0.001), but not with histological grade, molecular type and other Immunohistochemical parameters(ER, PR, HER2 and Ki-67). In a univariate survival analysis, tumor stage, tumor size, lymph node involvement (*p*<0.001/ *p*<0.001)and plasma fibrinogen (*p*<0.001/ *p*<0.001) levels were associated with disease-free and overall survival, but just lymph nodes involvement (*p*<0.001, hazard ratio [HR] = 2.9, 95% confidence interval [CI] = 1.6–5.3/ *p* = 0.006, HR = 3.2, 95% CI = 1.4–7.3) and plasma fibrinogen levels (*p* = 0.006, HR = 3.4, 95% CI = 1.4–8.3/ *p* = 0.002, HR = 10.1, 95% CI = 2.3–44.6) were associated with disease-free and overall survival in a multivariate survival analysis, respectively.

**Conclusions:**

This study demonstrates that elevated preoperative plasma fibrinogen levels are associated with breast cancer progression and are independently associated with a poor prognosis in patients with operable breast cancer.

## Introduction

Breast cancer is the most frequently diagnosed cancer and the leading cause of cancer death among females worldwide, with an estimated 1.7 million cases and 521,900 deaths in 2012, It alone accounts for 25% of all cancer cases and 15% of all cancer deaths among females[1]. The incidence rates and mortality rates are stable or decreasing in more developed countries but increasing in less developed countries. The lack of better adjuvant therapy is still the main cause of death in patients with recurrence and metastasis of breast cancer. Nowadays, the tumor, node, and metastasis (TNM) staging system of the American Joint Committee on Cancer (AJCC) has been broadly recognized, and the plasma cancer antigen 15–3 level has conventionally become a simple and clinically useful method for routine surveillance, diagnosis, and the evaluation of prognosis, but the worldwide recognized system or marker for preoperatively predicting the prognosis of patients with breast cancer is uncertain. And with the application of neoadjuvant chemotherapy and endocrine therapy, postoperative clinicopathological parameters often change, which influences the judgment of real prognosis. Therefore, a biological characteristics that can predict recurrence and metastasis is very important for patients with operable breast cancer, maybe it can develop the treatment plans of breast cancer.

The hypercoagulable state is deemed to be associated with malignancy which has showed a high record of 50% in cancer patients[2, 3]. Fibrinogen, a 340-kDa glycoprotein that is synthesized by liver and is converted to fibrin by activated thrombin, is an important coagulation factor. It is a modest acute-phase response protein that will increase in concentration in response to most forms of tissue injury, infection or inflammation[4, 5]. Recently, current studies showed that fibrinogen played vital roles in tumourigenesis and contributed to angiogenesis, stroma formation, and haematogenous metastasis of tumor cells[6–9]. In the meantime, a host of studies have indicated that elevated plasma fibrinogen levels were related with tumor progression and other poor outcomes in several different types of malignancies, covered esophageal cancer[10], gastric cancer[11], pancreatic cancer[12], colon cancer[13], lung cancer[14], hepatocellular cancer[15], gallbladder cancer[16], and gynecological cancer[17–20]. However, as far as we know, the clinical and prognostic value of preoperative plasma fibrinogen levels in patients with operable breast cancer is not been well defined.

Therefore, in this current study, we investigated the correlation between the preoperative plasma fibrinogen levels, clinicopathological parameters, the disease-free and overall survival rates in patients with operable breast cancer.

## Materials and Methods

### Patients

485 operable patients with invasive breast cancer treated at Qilu Hospital of Shandong University from January 2009 to January 2011 were prospectively enrolled in this study. All of medical records were reviewed retrospectively. Patients were included if they met the following criteria: (1) a newly diagnosed breast cancer (2) histological confirmation of diagnosis; and (3) willingness to participate. Exclusion criteria for all participants were: (1) overt bacterial or viral infection; (2) with liver disease; (3) blood coagulation disorders; (4) oral administration of anticoagulants or acetylsalicylic acids within 3 month before the treatment; (5) preoperative chemotherapy or radiotherapy; (6) no adequate clinical data or loss of follow up. In total, 223 patients were left to be evaluated for the study.

This study complied with the standards of the Declaration of Helsinki as well as with current ethical guidelines and was approved by the institutional ethical committee of the Qilu Hospital of Shandong University. Written, informed consent for participation in the study was obtained from all the patients.

### Plasma Fibrinogen Levels

Plasma fibrinogen concentrations of peripheral venous blood samples taken before breakfast were examined less than 7 days before surgery. For assaying fibrinogen, we used the clotting method of Clauss with the STA®-Fibrinogen ⑤ kit (DIAGNOSTICA STAGO Company, Ltd., Asnieres sur Seine, France). The intraassay coefficient and interassay coefficient of fibrinogen were both less than 5%. The reference ranges for plasma fibrinogen levels was defined as 2.0–4.0 g/l. Plasma fibrinogen levels higher than 4.0 g/l indicated hyperfibrinogenaemia.

### Clinical Treatment and Patient Follow-up

All patients were treated by modified radical mastectomy or radical mastectomy, and received systemic therapy in the adjuvant setting. Patients were followed carefully after the initial treatment and reassessed every 3 months for the next 3 years and once a year thereafter. The start of the follow-up was the date of the initial diagnosis of breast cancer. The end of the follow-up was the time of the last follow-up encompassed by this study (March 2015) or death.

### Statistics

Plasma fibrinogen levels are presented as means ± SD. Categorical variables are presented as numbers and percentages. Comparisons between groups were performed using the Kruskal-Wallis test and the Wilcoxon rank-sum test for continuous variables. Score test was used to estimate the sample size. The end points of the analyses were Disease-free survival (DFS) and Overall survival (OS). Disease-free survival was defined as the period from the date of initial diagnosis to the date of recurrence, metastasis or censoring. Overall survival was calculated as the time from initial diagnosis to death or censoring. The disease-free survival and overall survival rates were calculated using the Kaplan-Meier method, and differences in survival rates between the groups were compared by the log-rank test. For survival analysis, the optimal cutoff level for plasma fibrinogen levels was determined by a time-dependent receiver operating characteristics analysis. Both univariate and multivariate analyses were performed for the prognostic factors using the Cox proportional hazard model. We tested the proportional hazards assumption for the multivariable models. The internal stability of the models was tested by bootstrap resampling. Briefly, new data sets with the same size of the original one were created by random sampling with replacement. We calculated 1,000 Bootstrap samples from the original data set followed by the same Cox regression. The performance evaluation of the model was tested by residual analysis and model comparisons. A two-sided p <0.05 was considered to be statistically significant. Score test was performed using PASS 14.0.2(NCSS LLC, Kaysville, Utah, USA). Time-dependent receiver operating characteristics was analyzed by R from CRAN. The other data analysis was performed using SAS version 9.4(SAS Institute, Inc., Cary, NC, USA) (S1 File).

## Results

### Patient Characteristics

In this study, the median age of the patients was 47 years (range 27–79). The hyperfibrinogenaemia incidence was 17.9%. 79 patients (35.4%) were menopause. Building on the tumor staging system, most patients were diagnosed at stage Ⅱ (145, 65.0%) and 42 patients (18.8%) were stage Ⅲ. 121 patients (54.3%) had lymph node metastasis, of which 54 patients had at least 4 nodes involvement. Building on the Immunohistochemical analysis for ER, PR, HER2 and Ki-67, we calculated molecular subtypes: Luminal A (ER+ and/or PR+, HER2-, Ki67≤20%)(63, 28.3%), Luminal B (ER+ and/ or PR+, HER2-, Ki67>20%)(94, 42.2%), Basal-like (ER- and PR-, HER2-)(38, 17.0%), and HER2+ (ER+/-, PR+/-, HER2+)(28, 12.5%). No patients had local relapse or died from causes unrelated to breast cancer, 26 (11.7%) patients developed distant metastasis and 18 (8.0%) died from breast cancer, 205 (92.0%) patients were alive at the date of last follow-up. The baseline patient characteristics are shown in Table 1.

**Table 1:** Baseline characteristics of the patients.

Variable	Number	(%)
Age		
≤47	119	53.36
>47	104	46.64
Menopause		
No	144	64.57
Yes	79	35.43
Tumor stage		
I	36	16.14
II	145	65.02
III	42	18.84
Histological grade		
G1	10	4.48
G2	150	67.26
G3	63	28.26
Tumor size		
T1&T2	193	86.55
T3&T4	30	13.45
Lymph node involvement		
0	102	45.74
1~3	67	30.04
≥4	54	24.22
ER		
negative	66	29.60
positive	157	70.40
PR		
negative	83	37.22
positive	140	62.78
HER2		
negative	188	84.30
positive	35	15.70
Ki-67		
≤20%	92	41.26
>20%	131	58.74
Molecular Type		
Luminal A	63	28.25
Luminal B	94	42.15
HER2+	28	12.56
Basal-like	38	17.04
Metastasis		
Yes	26	11.66
No	197	88.34
Death		
Yes	18	8.07
No	205	91.93

### Correlation between Preoperative Plasma Fibrinogen Levels and Clinicopathological Parameters

**Fig 1:**
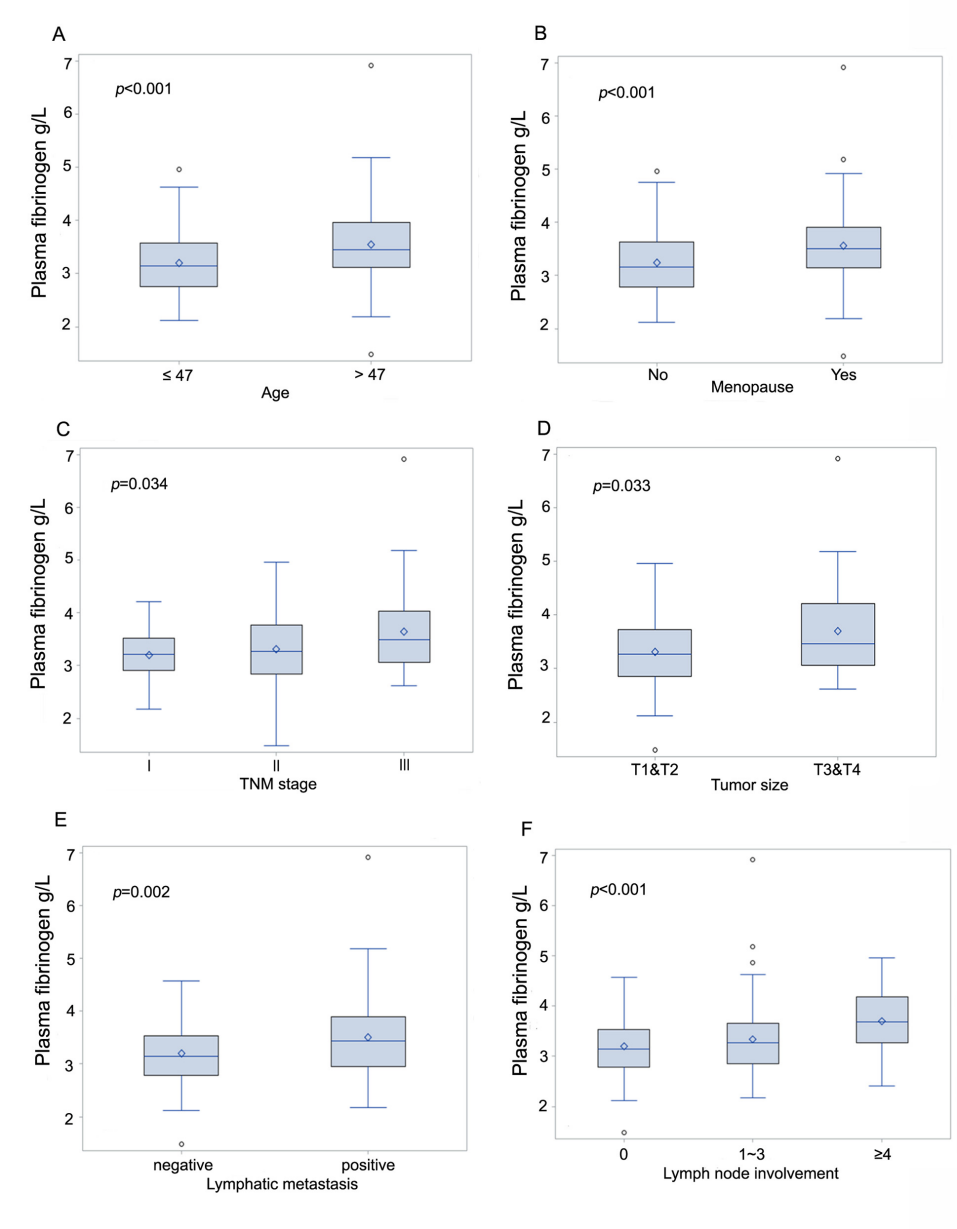
Box plots indicating the distribution of the pretreatment plasma fibrinogen levels and the following clinicopathological parameters: age (A), menopause (B), tumor stage (C), tumor size (D), lymphatic metastasis (E), and lymph node involvement (F). The horizontal bar in the box indicates the median value, the box indicates the 1st (25%) and 3rd (75%) quartile, the whiskers indicate 5–95%, the open circles indicate the outlier

The mean value of the preoperative plasma fibrinogen levels was 3.36 ± 0.69 g/l. Elevated fibrinogen plasma levels were associated with older age (*p* < 0.001,Fig 1A), menopause (*p* < 0.001,Fig 1B), advanced tumor stage (*p* = 0.034,Fig 1C), larger tumor size (*p* = 0.033,Fig 1D), lymphatic metastasis (p = 0.002,Fig 1E), and lymph node involvement (*p* < 0.001,Fig 1F), but not with histological grade (*p* = 0.543), ER (*p* = 0.966), PR (*p* = 0.422), HER2 (*p* = 0.466), Ki-67 (*p* = 0.720) or molecular type (*p* = 0.464). All the association is shown in Table 2.

**Table 2:** Correlation between preoperative plasma fibrinogen levels and clinicopathological parameters.

Variables	Number (%)	mean fibrinogen levels, g/l	P
Patients	223	3.36±0.69	
Age, years			**<0.001**
≤47	119 (53.36)	3.20±0.59	
>47	104 (46.64)	3.55±0.74	
Menopause			**<0.001**
No	144 (64.57)	3.25±0.63	
Yes	79 (35.43)	3.57±0.75	
Tumor stage			**0.034**
I	36 (16.14)	3.20±0.50	
II	145 (65.02)	3.32±0.67	
III	42 (18.84)	3.65±0.80	
Histological grade			0.543
G1	10 (4.48)	3.17±0.60	
G2	150 (67.26)	3.35±0.69	
G3	63 (28.26)	3.42±0.70	
Tumor size			**0.033**
T1&T2	193 (86.55)	3.31±0.64	
T3&T4	30 (13.45)	3.71±0.90	
Lymph node involvement			**<0.001**
0	102 (45.74)	3.19±0.59	
1~3	67 (30.04)	3.34±0.78	
≥4	54 (24.22)	3.70±0.63	
Lymphatic metastasis			**0.002**
negative	102 (45.74)	3.19±0.59	
positive	121 (54.26)	3.50±0.74	
ER			0.966
negative	66 (29.60)	3.35±0.65	
positive	157 (70.40)	3.36±0.71	
PR			0.442
negative	83 (37.22)	3.40±0.65	
positive	140 (62.78)	3.34±0.71	
HER2			0.466
negative	188 (84.30)	3.36±0.65	
positive	35 (15.70)	3.34±0.86	
Ki-67			0.720
≤20%	92 (41.26)	3.39±0.74	
>20%	131 (58.74)	3.34±0.65	
Molecular type			0.464
Luminal A	63 (28.25)	3.30±0.66	
Luminal B	94 (42.15)	3.38±0.66	
HER2+	28 (12.56)	3.31±0.90	
Basal-like	38 (17.04)	3.45±0.64	

### Survival Analysis

Score test was used to estimate the sample size. Based on a condition of establishing Cox proportional hazards model, when sample size was greater than or equal to 154 patients, the power of this test was larger than 0.8.

**Fig 2:**
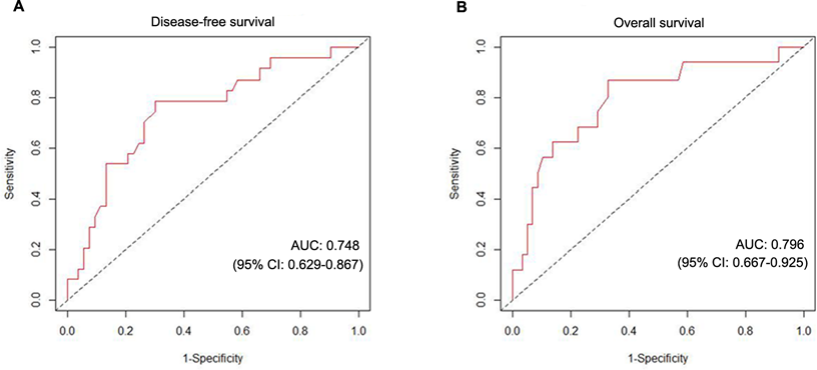
The time-dependent receiver operating characteristics analysis of the plasma fibrinogen level to predict metastasis and death in patients with operable breast cancer.

We used the time-dependent receiver operating characteristics analysis to investigate the sensitivity and specificity of plasma fibrinogen levels in predicting metastasis (DFS) and death (OS). The area under the curve (AUC) for prognosticating DFS and OS was 0.748 (95%CI 0.629–0.867) and 0.796 (95%CI 0.667–0.925) at time t = 60 month, respectively, indicating that plasma fibrinogen level had equally high accuracy for predicting DFS and OS in patients with operable breast cancer (Fig 2). With the highest Youden index, we determined the optimal cutoff value for the plasma fibrinogen level was 3.46 g/l. The sensitivity, specificity, positive predictive value, and negative predictive value of the cutoff value for DFS was 78.6% (95%CI = 52.2–88.4), 69.8% (95%CI = 57.9–71.6), 24.4% (95%CI = 13.5–31.6), and 96.3% (95%CI = 89.6–97.9), and for OS was 87.0% (95%CI = 65.3–98.6), 58.6% (95%CI = 57.9–71.4), 14.6% (95%CI = 10.8–27.8), and 98.2% (95%CI = 94.8–99.8), respectively.

The clinical reference ranges for plasma fibrinogen levels was defined as 2.0–4.0 g/l, levels higher than 4.0 g/l indicated hyperfibrinogenaemia. we categorized patients by two groups according to the mean value(fib = 3.36g/L) or fib = 4g/L, respectively, The Kaplan-Meier curves for DFS and OS of each groups had statistically significant differences (S1 Fig and S2 Fig), patients in the low-plasma fibrinogen group had dramatic longer DFS and OS than patients in the high-plasma fibrinogen group. Fib = 3.46g/l, the optimal cutoff value determined by the time-dependent receiver operating characteristics analysis, had higher accuracy for predicting DFS and OS than fib = 4g/L at 5 year long. So although 4g/L is a clinical cutoff value, patients with fib>3.46g/L need to be followed closely, we suggest to reassess with 3 months interval for the following 5 years. According to the cutoff level, we divided patients into two groups (≤ 3.46 g/l, n = 135; >3.46 g/l, n = 88).

**Fig 3:**
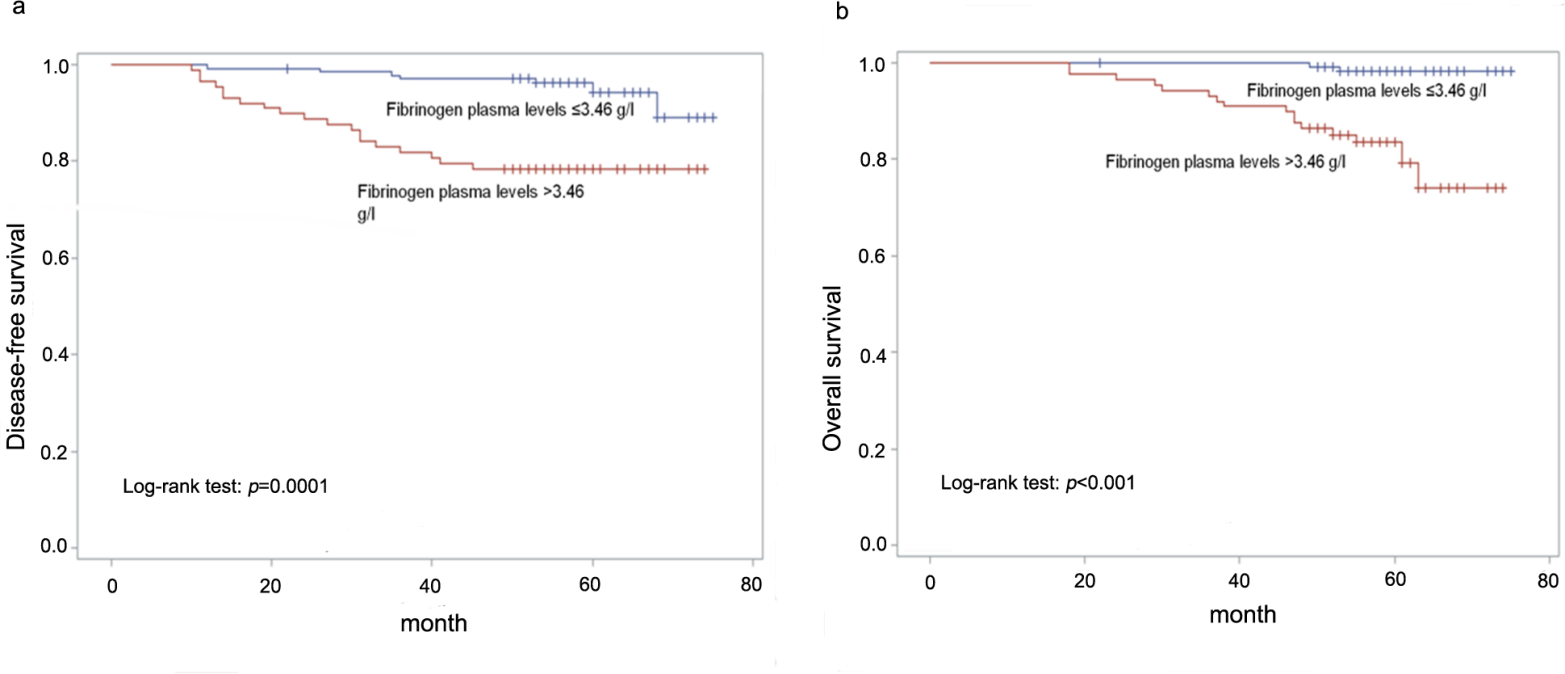
A, B Kaplan-Meier curves for DFS and OS according to plasma fibrinogen levels (curves were separated by the cutoff value).

The median follow-up duration was 56 months (range 10–75 (DFS) and 18–75 (OS)). To assess the prognostic impact of preoperative plasma fibrinogen levels, we performed Kaplan-Meier analysis to compare patients grouped according to the cutoff value, The 3- and 5-year disease free survival rates and overall survival rates in patients were 91.0%/97.3% (DFS/OS) and 88.8%/92.8%, plasma fibrinogen levels ≤3.46 g/l were 97.0%/100% (DFS/OS) and 95.6%/98.5%, respectively. These survival rates in the other group were 81.8%/93.2% and 78.4%/84.1%, respectively. Patients in the low-plasma fibrinogen group (≤3.46 g/l) had longer DFS and OS than patients in the high-plasma fibrinogen group (> 3.46 g/l), respectively (*p* = 0.0001, *p*<0.001, Fig 3).

### Prognostic Factor

We tested the proportional hazards assumption for the multivariable models and the plasma fibrinogen levels must be used as categorical variable (categorized according to the cutoff value) in our multivariate Cox regression Model.

In the univariate analysis, tumor stage, tumor size, lymph node involvement and plasma fibrinogen levels were associated with both DFS and OS (Table 3). Then when we used the multivariate analysis to verify the potential prognostic factors, just the plasma fibrinogen levels and the lymph node involvement were found to be independently associated with DFS and OS.(Table 3). The internal stability of the models was tested by bootstrapping. When the difference between the estimates of parameters calculated by bootstrap method and original data less than 25% of the standard error calculated by bootstrap sampling, bias of parameter estimation can be negligible. Only the model of DFS met the requirements, the histograms for OS showed relatively long tails on the right side. The short time follow-up potentially lead to this.

**Table 3:** Univariate and multivariate survival analysis in patients with operable breast cancer.

	Disease-free survival				Overall survival			
	univariate		multivariate		univariate		multivariate	
	p	HR(95%CI)	p	HR(95%CI)	p	HR(95%CI)	p	HR(95%CI)
Age(vs.≤47)	0.405	0.7(0.3–1.6)	n.s.		0.524	0.7(0.3–1.9)	n.s.	
Menopause(vs.no)	0.188	0.5(0.2–1.3)	n.s.		0.519	0.7(0.3–2.0)	n.s.	
Tumor stage(Ⅰvs.Ⅱvs.Ⅲ)	<0.001	3.1(1.6–6.1)	0.939	n.s.	<0.001	4.3(1.9–9.9)	0.649	n.s.
Plasma fibrinogen, g/l	<0.001	4.7(2.0–11.2)	0.006	3.4(1.4–8.3)	<0.001	13.3(3.1–58.0)	0.002	10.1(2.3–44.6)
Histological grade (G1 vs. G2 vs.G3)	0.734	1.1(0.5–2.4)	n.s.		0.900	0.9(0.4–2.3)	n.s.	
Tumor size(T1 &T2 vs. T3&T4)	<0.001	4.0(1.8–9.0)	0.061	n.s.	0.002	4.5(1.7–11.6)	0.118	n.s.
Lymph node involvement	<0.001	3.7(2.1–6.5)	<0.001	2.9(1.6–5.3)	<0.001	4.7(2.2–10.4)	0.006	3.2(1.4–7.3)
ER	0.379	1.5(0.6–3.8)	n.s.		0.209	2.2(0.6–7.6)	n.s.	
PR	0.720	1.2(0.5–2.6)	n.s.		0.742	1.2(0.4–3.1)	n.s.	
HER2	0.801	0.9(0.3–2.5)	n.s.		0.200	0.3(0–2.0)	n.s.	
Ki-67	0.899	1.1(0.5–2.3)	n.s.		0.680	0.8(0.3–2.1)	n.s.	
Molecular Type	0.761	1.1(0.7–1.5)	n.s.		0.706	0.9(0.6–1.5)	n.s.	

n.s. = Not significant; CI = confidence interval; HR = hazard ratio.

## Discussion

In recent years, a slice of studies confirmed that elevated plasma fibrinogen levels are associated with tumor progression and poor prognosis in several types of cancer[21–24]. In our study, preoperative plasma fibrinogen levels are associated with a multitude of risk factors of breast cancer, including age, menopause or not, tumor size, lymph node involvement and tumor staging, but not with histological grade, immunohistochemical parameters and molecular type. Michael demonstrated that human aging increased the plasma concentration of fibrinogen, although the possible causes that explain this phenomenon were complex[25]. So the correlation between age (or menopause) and the plasma fibrinogen levels of patients with breast cancer has no special significance. Elevated preoperative plasma fibrinogen levels were associated with larger tumor size, TNM staging and more lymph nodes metastasis, which demonstrate that plasma fibrinogen levels are closely related to tumor progression and metastasis. In addition, multivariate survival analyses showed that preoperative plasma fibrinogen levels and lymph node involvement are independent prognostic factors for poor survival in patients with operative breast cancer. Elevated preoperative plasma fibrinogen levels (especially > 3.46 g/l) can indicate earlier metastasis or death.

The levels of fibrinogen increase in response to most physiological and pathological conditions, such as acute infection, tissue injury, shock, hypercoagulable state, acute myocardial infarction and malignant tumor[4]. The procedure is cytokine driven through IL-6 and others, which responsible for the acute-phase changes usually seen upon infection or injury[26, 27]. To our knowledge, the reason for the association between elevated plasma fibrinogen levels and the postoperative pathological parameters of cancer, and whether this affect tumor progression, remains unknown. Quite a few researches show that elevated plasma fibrinogen levels may be induced by an inflammatory reaction to tumor growth and a hypercoagulable state in cancer patients[2, 19, 28]. Other studies show that fibrinogen can be endogenously synthesised by tumor cells themselves[6, 29]. Studies on the molecular level show that through interactions with fibroblast growth factor-2 (FGF-2) and vascular endothelial growth factor (VEGF), fibrinogen can regulate cellular adhesion, proliferation, and migration[5, 30]. Shu demonstrated that highly concentrated fibrinogen induced Epithelial-Mesenchymal Transition (EMT) by increasing the expression of vimentin and reducing expression of E-cadherin[16]. EMT confers migration, invasion, and metastatic capacity, and multidrug resistance to tumor cells[31, 32]. All of the above can explain our present study, tumor growth and local invasion lead to inflammation which increase the plasma fibrinogen levels, and elevated fibrinogen levels facilitate the stable adhesion and survival of metastatic emboli after tumor cell intravasation, it may be the reason of the hematogenous and lymphatic metastasis of cancer.

This study demonstrated the clinical and prognostic significance of preoperative plasma fibrinogen levels in patients with operable breast cancer for the first time. The detection of preoperative plasma fibrinogen levels is a routine examination of patients, which has the advantage of being inexpensive and readily available, and the levels can be measured repeatedly. In recent studies, pretreatment plasma C-reactive protein and D-dimer levels are also found to be predictive biomarkers in breast cancer patients[33–35]. Due to the application of the neoadjuvant chemotherapy in breast cancer patients, clinicopathological parameters such as tumor size, lymph node metastasis, tumor staging and postoperative immunohistochemical markers will change, then these preoperative plasma biomarker levels can be applied to the prognosis assessment of patients with breast cancer.

The recent animal experiment demonstrated that spontaneous hematogenous and lymphatic metastasis was diminished in fibrinogen-deficient mice. This research suggested that therapeutic strategies focusing on hemostatic factors may be effective in controlling solid tumor metastasis, particularly used for the treatment of micrometastatic disease[30]. Also other clinical trials have suggested that anticoagulants, such as low-molecular-weight heparins (LMWHs) and heparins, can improve the survival of cancer patients[36]. Although further clinical studies are needed to determine the efficacy and safety of anticoagulants, it should be noted that anticoagulation therapy is a promising treatment strategy for improving prognosis in patients with cancer.

There were some limitations to this study: single medical center, small number of participants, retrospective research and short-time follow-up potentially lead to an inappropriate conclusion. Therefore, a largescale multi-center prospective validation study with longer follow-up period is needed to further determine the results.

This study demonstrates that elevated preoperative plasma fibrinogen levels are associated with tumor progression and are independently associated with a poor prognosis in patients with operative breast cancer. Fibrinogen is a novel independent prognostic biomarker and potential therapeutic target in breast cancer patients.

## Supporting Information

S1 Fig a, b Kaplan-Meier curves for DFS and OS according to plasma fibrinogen levels (curves were separated by the mean value). (PDF)

S2 Fig a, b Kaplan-Meier curves for DFS and OS according to plasma fibrinogen levels (curves were separated by the clinical cutoff value). (PDF)

S1 File The SAS log file of detailed statistical analysis procedures. (LOG)
