# Supplementary figures and images for: Clinical and Prognostic Significance of Preoperative Plasma Fibrinogen Levels in Patients with Operable Breast Cancer
Source: PLoS One. 2016 Jan 22;11(1):e0146233. doi: 10.1371/journal.pone.0146233 (PMC4723094; doi:10.1371/journal.pone.0146233)

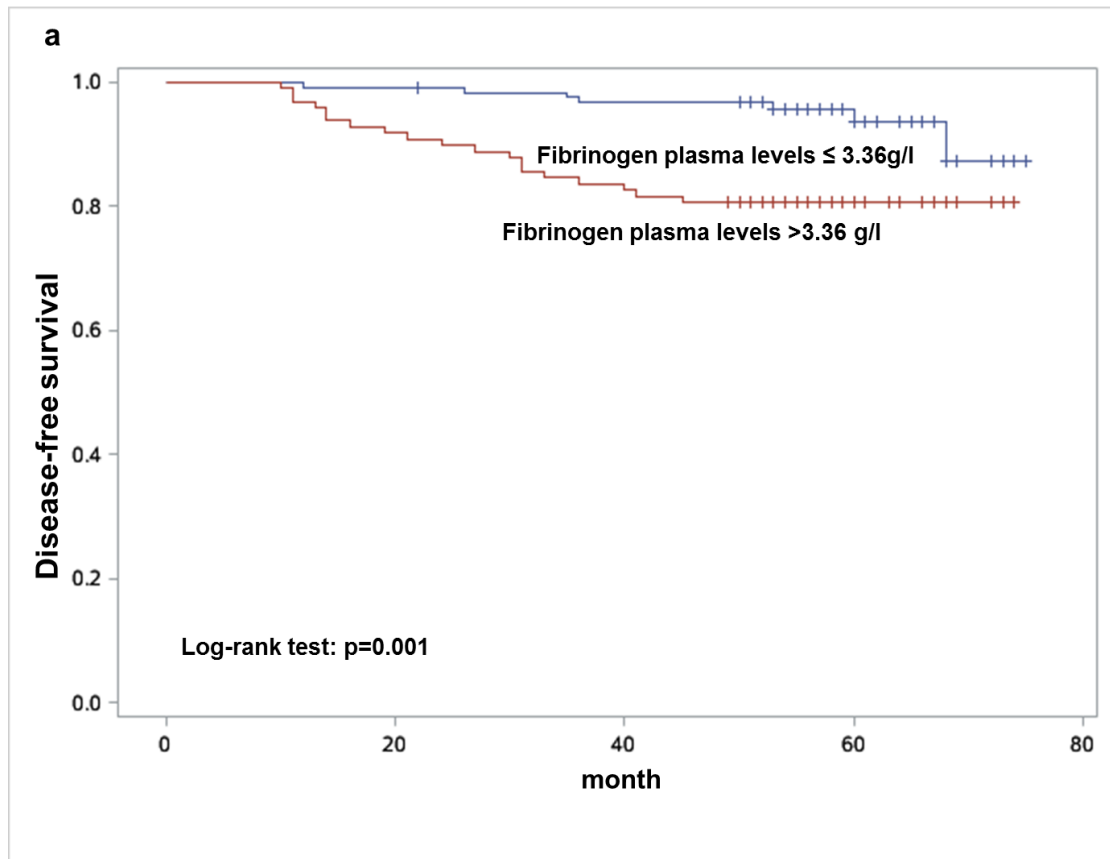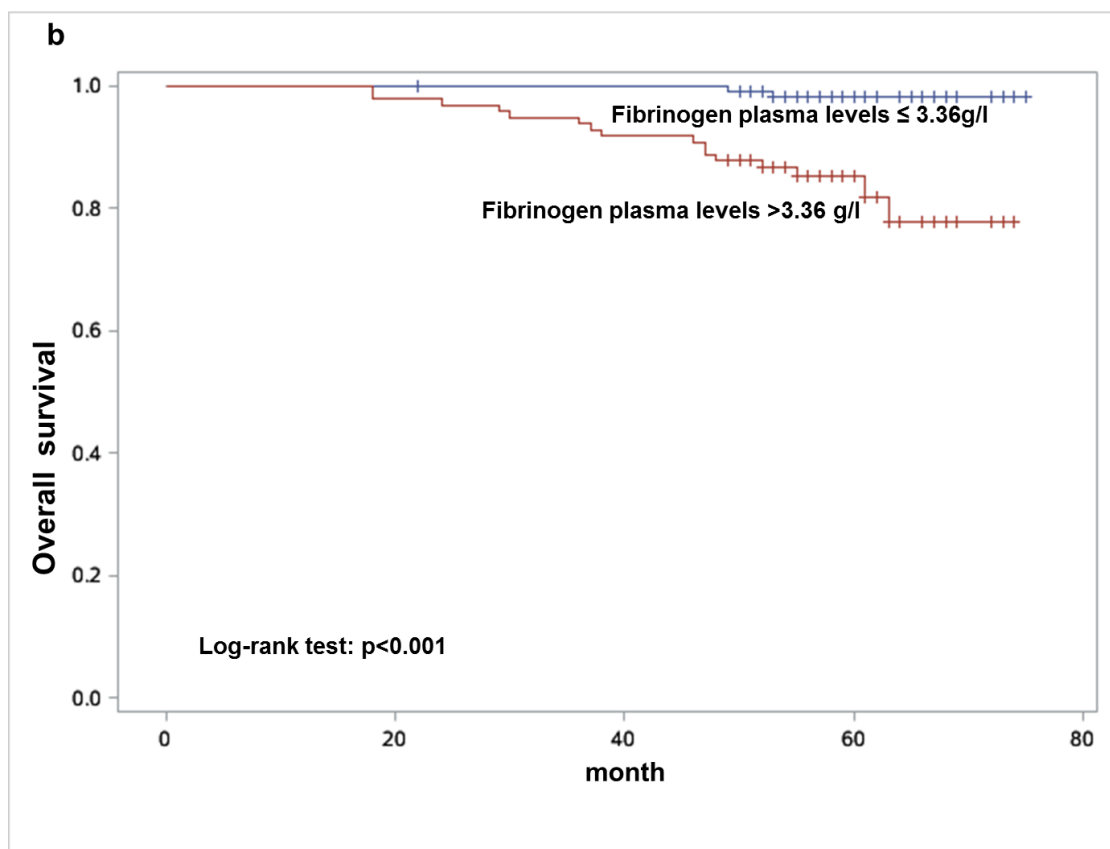

Supplement: S1 Fig — (PDF) [file pone.0146233.s001.pdf]

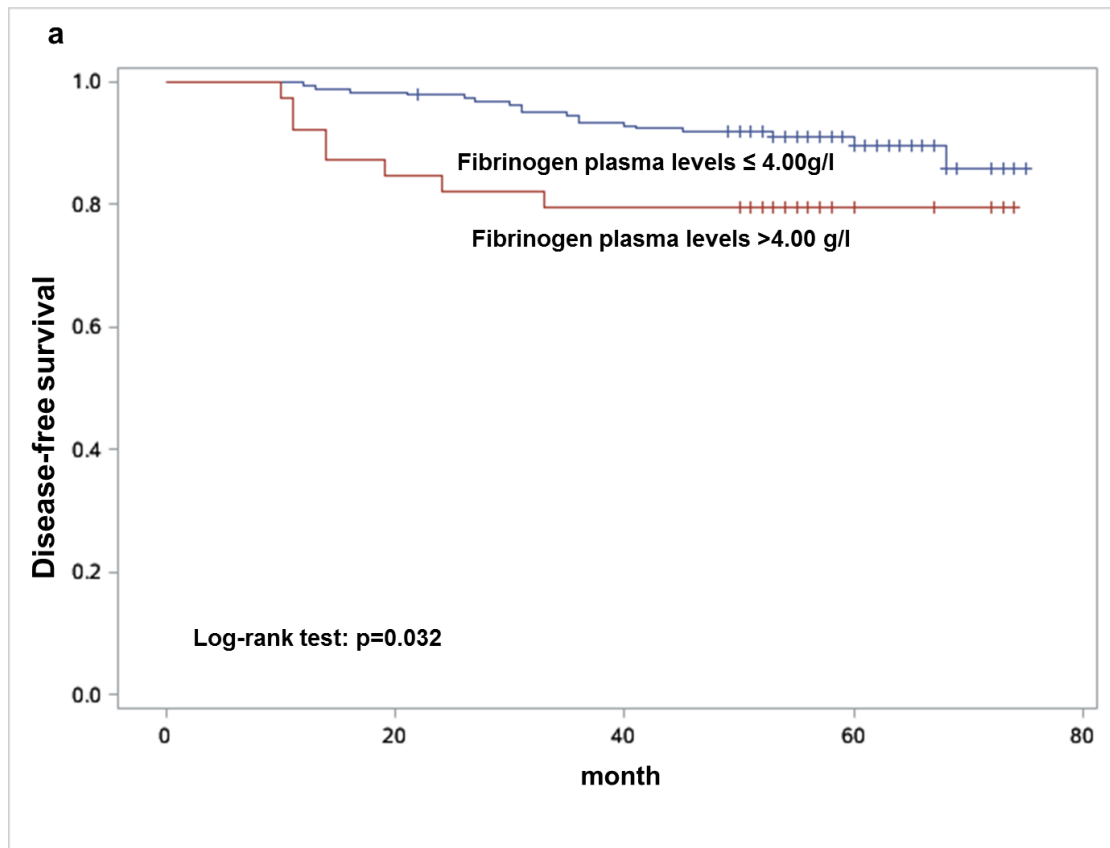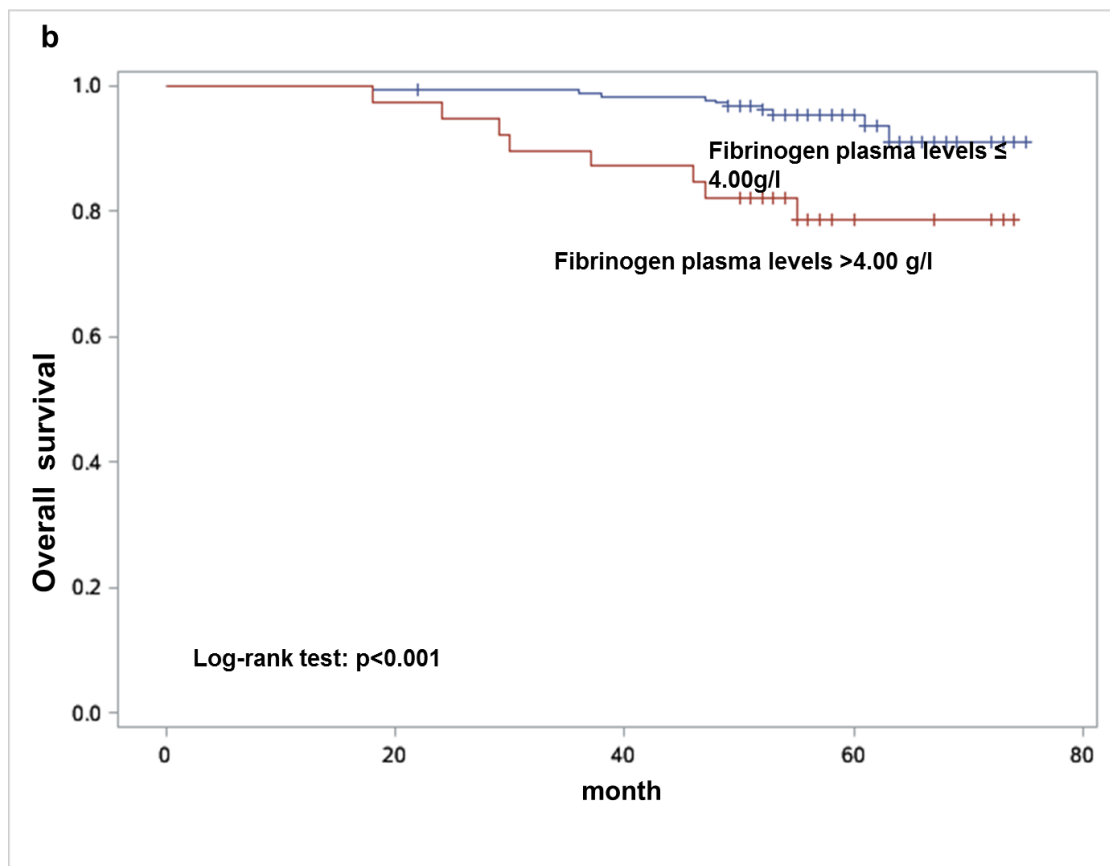

Supplement: S2 Fig — (PDF) [file pone.0146233.s002.pdf]
